# Cardioprotective Effect of Decorin in Type 2 Diabetes

**DOI:** 10.3389/fendo.2020.479258

**Published:** 2020-12-07

**Authors:** Fuqiong Chen, Jinsheng Lai, Yanfang Zhu, Mengying He, Huiying Hou, Jin Wang, Chen Chen, Dao Wen Wang, Jiarong Tang

**Affiliations:** ^1^ Department of Endocrinology, Tongji Hospital, Tongji Medical College, Huazhong University of Science and Technology, Wuhan, China; ^2^ Division of Cardiology, Department of Internal Medicine, Tongji Hospital, Tongji Medical College, Huazhong University of Science and Technology, Wuhan, China; ^3^ Hubei Key Laboratory of Genetics and Molecular Mechanisms of Cardiological Disorders, Tongji Hospital, Tongji Medical College, Huazhong University of Science and Technology, Wuhan, China; ^4^ Department of Cardiology, Zhongnan Hospital of Wuhan University, Wuhan, China

**Keywords:** fibrosis, inflammation, gene therapy, decorin, diabetic cardiomyopathy

## Abstract

Cardiomyopathy is the leading cause of increased mortality in diabetes. In the present study, we investigated the effects of decorin (DCN) gene therapy on left ventricular function, cardiac inflammation and fibrosis in type 2 diabetes. Type 2 diabetes was induced in male Wistar rats by high fat diet (HFD, 60% of calories as fat) and STZ (20 mg/kg, intraperitoneal). Diabetic rats were divided into (n=6 for each group) the control group, the GFP-treated group and the DCN-treated group, received intravenous injection of saline solution, recombinant adeno-associated viral (rAAV)-GFP, and rAAV-DCN, respectively. We evaluated cardiac inflammation, fibrosis, left ventricular function at 6 months after gene delivery. Results turned out that rAAV-DCN treatment attenuated diabetic cardiomyopathy with improved LV function compared with control animals, which might be related to the reduced cardiac inflammation and fibrosis. These protective effects were associated with TGFβ1 pathway (ERK1/2 and smad-2) and NF-κB pathway, which may due to the decreased activation level of IGF-IR, increased expression of PKC-α and Hsp70. In conclusion, our results show that rAAV-mediated DCN therapy may be beneficial in the treatment of Diabetic Cardiomyopathy.

## Introduction

Diabetic cardiomyopathy (DCM) is one of the major causes of increased mortality in patients with diabetes mellitus (DM) (1). It is defined as left ventricular systolic and (or) diastolic dysfunction independent of the traditional risk for HF, including hypertension, coronary heart disease and valvular heart disease (2). Previous studies showed that diverse pathogenic mechanisms are involved in DCM, including excessive composition of the extracellular matrix with enhanced cardiac fibrosis (3), cardiac inflammation characterized by increased levels of pro-inflammatory cytokines, coupled with activation of mitogen-activated protein kinases (MAPKs) and nuclear factor (NF-κB) (4).

Decorin (DCN) is a member of the small leucine-rich proteoglycan (SLRP) gene family characterized by core proteins with leucine-rich repeats (5, 6). Numerous studies have shown that DCN is able to regulate cellular functions by fettering to extracellular matrix (ECM) molecules or through receptors in cell surface, such as transforming growth factor beta (TGF-β), epidermal growth factor receptor (EGFR), insulin-like growth factor-1 receptor (IGF-1R), vascular endothelial growth factor receptor 2 (VEGFR2) (7). Thereby, DCN has multiple beneficial effects, such as anti-fibrogenesis, anti-inflammation, inhibiting tumor growth and metastatic spreading (8).

Recent study revealed that diabetic nephropathy was meliorated by DCN (9), which was reported as a protective agent against diabetic nephropathy by inhibition of apoptosis and fibrosis (10). In the cardiovascular system, DCN deficiency results in larger infarct size, enhanced ventricular remodeling and reduced ventricular function in DCN-/- mice 2 weeks after acute myocardial infarction (11). Furthermore, in Spontaneously Hypertensive Rats, DCN overexpression therapy by recombinant adeno-associated viral (rAAV) vector can attenuate hypertension-induced cardiac fibrosis and improve cardiac function (12). Our previously study also revealed that DCN promoted angiogenesis in diabetic hearts (13).

DCN is one of the components of extracellular matrix. To deliver the target genes into heart tissue, several vectors could be employed to achieve it, such as plasmid, viral vector and so on. Recently, the rAAV vector is predominantly applied in the field of gene therapy because of its advantages in gene therapy applications, including low immunogenicity and genotoxicity, broad tissue tropism and high transduction efficiency *in vivo*, and long-term transgene expression (14). In the current study, rAAV serotype 9 was used to overexpress DCN in the heart, and the effects of DCN gene delivery in the pathogenesis of DCM were evaluated. Our results demonstrate that DCN attenuates left ventricular dysfunction, cardiac remodeling, and inflammation.

## Materials and Methods

### Cell Culture

293T cell were obtained from the American Type Culture Collection (Virginia, USA) and maintained in Dulbecco’s modified Eagle’s medium (DMEM) (Invitrogen, California, USA) with 10% fetal bovine serum (FBS) at 37°C in the presence of 5% CO2 at constant humidity.

### rAAV Plasmid Construction and rAAV Packaging

The rAAV vector plasmid carrying the green fluorescent protein (pxxUF1-GFP), the AAV serotype 9 packaging plasmid (pxx9) and the Ad helper plasmid were obtained from Professor Xiao xiao (Eshelman School of Pharmacy, University of North Carolina at Chapel Hill, Chapel Hill, NC, USA). In order to obtain the rAAV-DCN vector plasmid, the human DCN gene cDNA fragment (1080bp) containing the open reading frame was cloned. The cDNA fragment and the pxxUF1-GFP plasmid was treated with DNA restriction endonuclease NotI (Transgen Biotech, Beijing, China) according to the instructions. The fragments were recycled and linked to each other with the T4 DNA ligase (Thermo, MA, USA). The constructions were introduced into the E coli. bacteria by heat shock. Following, the bacteria was recovered with LB medium in the absence of antibiotics and subjected to the solid LB medium with ampicillin. 24 h later, the monoclonal bacterial colony which have taken up the plasmid were separated and amplified. The plasmid was extracted and sequenced using the cytomegalovirus (CMV) promoter primer. Finally, the construction carrying the right DCN open reading frame was used in the following experiments.

The rAAV-DCN and rAAV-GFP were packaged with the triple-plasmid co-transfection method in 293 cell lines, as described previously by Professor Xiao xiao (15). In brief, 2 h before transfection, 293 cell was cultured with fresh Dulbecco medium (Gibco, NY, USA) containing 10% fetal bovine serum (HyClone, UT, USA). The three plasmid DNA mixture (vector plasmid: pXX9 plasmid: Ad helper plasmid, 2:1:3) was dissolved in 1 ml of 0.25 M CaCl2 and then quickly mixed with 1 ml of HEPES-buffered saline (50 mM HEPES, 280 mM NaCl, 1.5 mM Na2HPO4) and added to the cells. 12 h later, the medium was replaced with fresh Dulbecco modified Eagle medium (Gibco, NY, USA) containing 10% fetal bovine serum. The cell was harvested at 48h after transfected. The rAAV vectors was obtained and purified with CsCl density gradient purification (15).

### Animal Treatment

Male Wistar rats (8 weeks old) were purchased from the Experimental Animal Center of Tongji Medical College, Huazhong University of Science and Technology. Animal housing rooms were maintained at a constant room temperature (25°C) and rats were exposed to a 12-h light/dark cycle. All animal procedures used in this study conformed to the National Institutes of Health (NIH) guidelines for the Care and Use of Laboratory Animals and were approved by the Chinese Academy of Sciences (Beijing, China). Type 2 diabetes was induced by high-fat diet (HFD D12492, 60% of calories as fat) and injection of low-dose STZ. After 1 month of HFD, the rats were injected with STZ (20 mg/kg, intraperitoneal). Meanwhile, the normal rats were treated with normal food (9% fat, 20% protein, 53% starch, 5% fiber) and injected with vehicle (citric acid, 50 mmol/L citrate buffer, pH 4.5). Four weeks after STZ injection, animals showing a fasting glucose level > 200 mg/dl were considered diabetes mellitus (DM) and were included in the experiments.

### Gene Delivery

The DM rats were randomly divided into three groups (n=6) as follow: the control group (treated with saline solution intravenously), the GFP-treated group (received rAAV-GFP vector) and the DCN-treated group (received rAAV-DCN vector). The normal diet-fed animals were used as non-diabetic controls. They were also randomly divided into three groups (the control group, the GFP-treated group and the DCN-treated group). Rats were injected with vehicle (1 ml of saline solution) or 1×10^11^/kg vector *via* the tail vein. All animals were sacrificed in 6 months after rAAV delivery under anesthetization. Organs were harvested, snap frozen in liquid nitrogen and stored at -80°C for later measurements and portion of the tissues was fixed with formalin for histological analysis.

### Blood Sample Collection and Measurements

Rats were fasted for 8 h. Approximately 0.5 ml blood was obtained from the tail vein using aseptic procedure every three weeks. Plasma was separated by centrifuge at 3,500 g for 8 min, and then stored at -80°C for later measurements. The blood glucose level was determined using an AEROSET Clinical Chemistry System (Abbott Laboratories, Illinois, USA). The blood insulin level was assayed by enzyme immunoassay with a rat insulin ELISA kit (MILLIPORE, MA, USA) according to the instruction.

### Oral Glucose Tolerance Tests

Rats were fasted for 12–16 h before oral glucose tolerance tests (OGTT). Rats were fed with 2.0 mg/g body weight glucose. Blood samples were collected from the tail at 0, 30, 60, 90, and 120 min after glucose administration. Plasma was obtained and the blood glucose was measured as aforementioned.

### Echocardiography and Hemodynamic Measurements

After the 6 months treatment period, echocardiography study was performed using an echocardiographic machine (8.5-MHz linear transducer; EnVisor C, Philips Medical Systems, Andover, MA, USA) to evaluate cardiac function. Rats were anesthetized with intraperitoneal injection of pentobarbital at a dose of 50 mg/kg body weight. M-mode tracings derived from parasternal long and short axis views were recorded to generate two-dimensional cines of the left ventricle. Left ventricular internal dimension of end-diastolic (LVIDd) and end-systolic diameters (LVIDs), intraventricular septum and posterior wall thickness on LV end-diastolic and end-systolic (IVS-D, LVD-PW, LVS-S, LVS-PW), LV fractional shortening (FS%) as well as ejection fraction (EF%) were measured.

In addition, cardiac catheterization studies were carried out. The pressure catheter (Millar-Instruments, Houston, TX) was induced into the left ventricle (1). Indexes of LV systolic and diastolic function were obtained from pressure-volume data at stabilization state. Indices were measured using analysis software Powerlab 7.0 (ADInstruments, Bella Vista, NSW, Australia), including the cardiac output (CO) (ml/min), heart rate (bpm), LV end systolic pressure (LVESP, mmHg), peak rates of LV pressure increased (dP/dt max), LV end diastolic pressure (LVEDP, mmHg), and peak rates of LV pressure decreased (dP/dt min; mmHg/s).

### Real-Time RT-PCR

Total RNA was isolated from frozen tissue samples with Trizol Reagent Kit (Invitrogen, CA, USA) according to the manufacturer’s instructions. All reagents for real-time RT-PCR were purchased from TransGen Company (Beijing, China). cDNA was prepared with the EasyScript First-Strand cDNA Synthesis SuperMix kit and real time PCR was performed with the TransStart Eco Green qPCR SuperMix kit (TransGen, Beijing, China) according to the recommendations of manufacturer. The mRNA level of IL 1α, IL 1β, IL 6, MCP-1, TGF-β1, Col 1A1, Col 1A2, Col 3A1, DCN, and GAPDH were detected. PCR amplification was performed using the specific primers. The specific primers were obtained from AUGCT Company (AUGCT, Beijing, China) and presented in **Supplementary Table 1**. The PCR were amplified as following: after an initial denaturation at 95°C for 30 s, amplification was finished by denaturing at 95°C for 5 s, annealing at 55°C for 15 s, and extending at 72°C for 10s through 40 cycles.

### Protein Extraction and Western Blots

The protein concentration was measured using the BCA protein assay reagent kit ((Boster Biotech, Wuhan, China). Western blot was performed as described previously (16). For detection of DCN, the samples were treated with chonchodinase ABC (Sigma Aldrich,  MO, USA) for 2h. To investigate the effects of rAAV-DCN treatment on cardiac tissue protein expression, blots were probed with specific antibodies against DCN, GFP, pERK, T-ERK, p-smad2, T-smad2, IGFR-1α, p-PKC-α, T-PKC-α, Hsp70, p-IκB, T-IκB, NFκB-p65, and β-actin. The information of the antibodies were presented in **Supplementary Table 2**.

### Enzyme-Linked Immunosorbent Assay

Plasma levels of IL-1α, IL-1β, IL-6, TGF-β1, and MCP-1 were determined in all the animals, using enzyme-linked immunosorbent assay (ELISA) kit following the manufacturer’s recommendations (Boster Biotech, Wuhan, China). Each sample was analyzed in duplicate, and the results were expressed as mean ± SD.

### Histological Studies

Cardiac tissues were fixed in 3.7% formalin, embedded in paraffin, and serially cut into 4 μm thick sections. For each animal, two sections were used for the different staining. To determine the degree of collagen fiber accumulation, sections were stained with Picrosirius Red satin solution (Boster Biotech, Wuhan, China) for 1 h at room temperature. HE staining was carried out with the HE staining Kit (Boster Biotech, Wuhan, China) according to the manufacture’s instruction. The sections were scanned at ×200 magnification. All stained sections were observed by two independent pathologists in a blind manner.

### Statistical Analysis

Data are presented as mean ± SD from at least three separate experiments. The data of blood glucose was analyzed by repeated measures analysis of variance (ANOVA) and other data were statistically analyzed using one-way ANOVA. When the results showed statistically significant, the LSD post-hoc test was used for further analysis. P value < 0.05 was considered as significant for all tests.

## Results

### RAAV-DCN Mediated Long-Term Overexpression of DCN *In Vivo*

**Figure 1:**
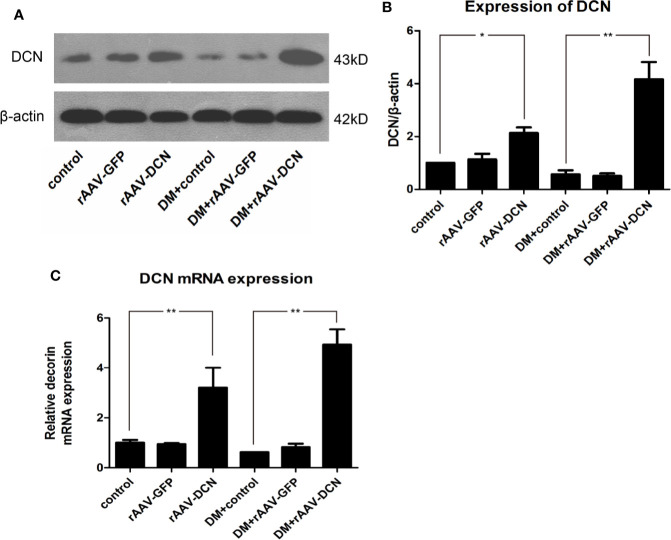
The recombinant adeno-associated viral vector mediated decorin (DCN) expression in heart tissue both in protein and mRNA level. **(A, B)** Western blot analysis showed that the expression levels of DCN protein was upregulated in the group received rAAV-DCN. **(C)** Real-time RT-PCR showed that DCN mRNA was significantly increased after rAAV-DCN administration. Values are means ± SD. *P < 0.05, **P < 0.01.

The efficiency of rAAV used in the experiment was evaluated by detection of GFP (**Supplementary Figures 1A, B**). Results showed that rAAV mediated the expression of GFP significantly, which mainly located in the cardiomyocytes. To evaluate the expression of DCN *in vivo*, total RNA and proteins were extracted from hearts. And level of DCN mRNA and protein were examined by real-time RT-PCR and Western blot analysis, respectively. As shown in **Figures 1A–C**, both were downregulated in diabetic rats, while DCN protein was significantly up-regulated in rAAV-DCN group than that in control (**Figures 1A, B**). Similarly, abundant DCN mRNA expression in heart tissues of rAAV-DCN-treated rats was observed compared to the control rats (**Figure 1C**). These results suggested that DCN mRNA and protein were stably expressed in the tissues after rAAV-DCN administration *in vivo*.

### Effect of DCN on Glucose Metabolism and Lipid Metabolism

**Figure 2:**
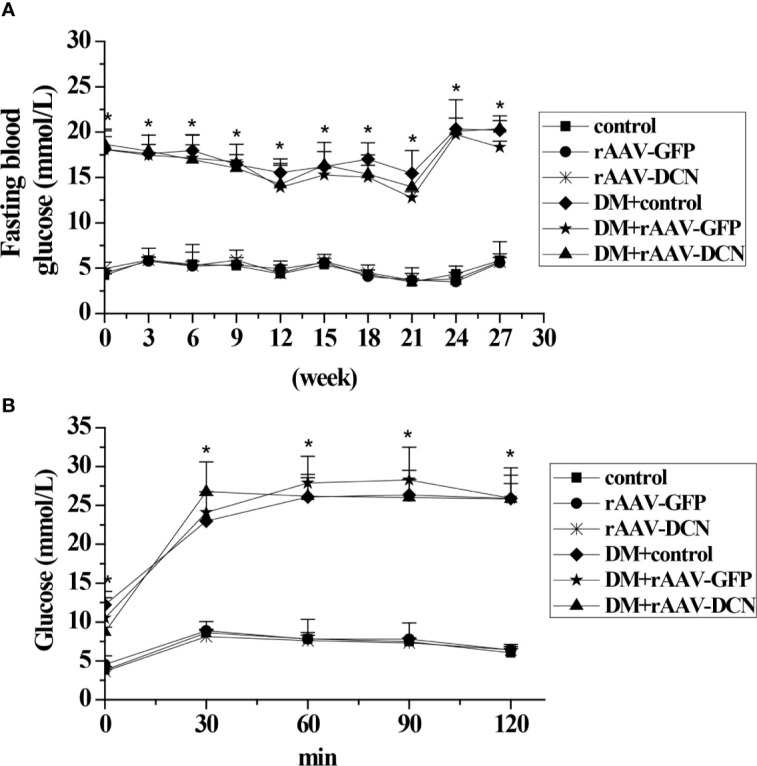
Effect of rAAV-decorin (DCN) treatment on glucose metabolism. **(A)** Fasting blood glucose were measured every two weeks throughout the study. **(B)** Plasma glucose levels during oral glucose tolerance tests (OGTT) after 6 months treatment. Values are means ± SD. *P < 0.05 diabetes mellitus (DM) group vs. control group.

In the present study, the blood sample was collected every three weeks and the blood glucose level was measured. As shown in **Figure 2A**, DCN showed no effect on blood glucose level. In addition, at the end of the study, OGTT was carried out to assess glucose tolerance. As expected, diabetic groups showed a higher level of fasted blood glucose than the control groups before the test. Moreover, after glucose administration, the blood glucose level of the diabetic groups was increased rapidly and maintained a high level in the next 2 h (about 25 mmol/L), while the control group increased less (peak level 10 mmol/L at 30 min) and decreased gradually. Nevertheless, Results showed that the curve of blood glucose of DM+rAAV-DCN group was similar to that of DM group. It meant that DCN overexpression had no effects on glucose tolerance (**Figure 2B**).

Besides, the plasma triglyceride (TG), total cholesterol (TC), low-density lipoprotein (LDL) and high-density lipoprotein (HDL) levels were measured after 6 months of rAAV-DCN injection. As expected, plasma TG in the DM groups were significantly elevated compared with non-diabetes controls, while rAAV-DCN treatment effectively decreased plasma TG level (**Supplementary Figure 2A**). However, TC, LDL and HDL levels were not significantly increased in the type 2 diabetes, which was comparable in rAAV-DCN treated groups (**Supplementary Figures 2B–D**).

### rAAV-DCN Attenuated Cardiac Dysfunction in DCM

**Figure 3:**
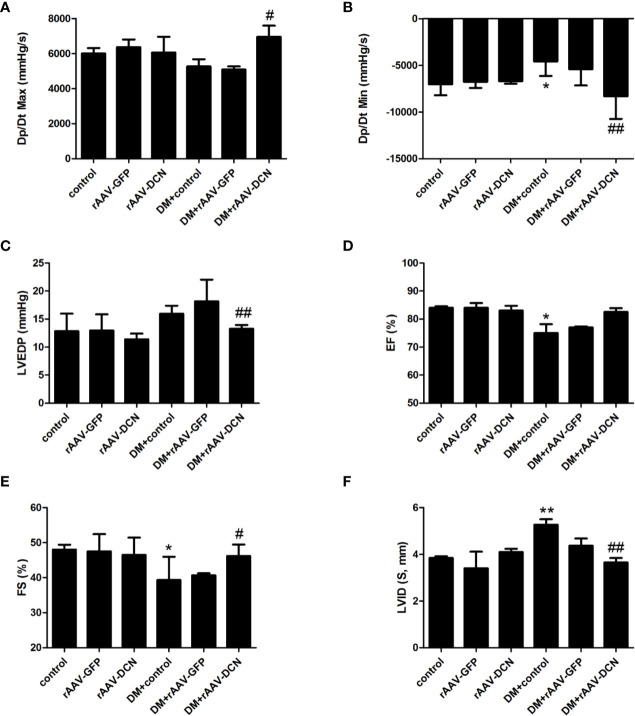
Overexpression of decorin (DCN) improved cardiac dysfunction in the diabetic rats. **(A)** The Dp/Dt maximum level (Dp/Dt max) evaluated by cardiac catheter; **(B)** The Dp/Dt minimum level (Dp/Dt min) evaluated by cardiac catheter; **(C)** End-diastolic pressure of left ventricular (LVEDP) evaluated by cardiac catheter; **(D)** Ejection fraction (EF) evaluated by echocardiography; **(E)** Fractional shortening (FS) evaluated by echocardiography; **(F)** left ventricular internal dimension (LVID) evaluated by echocardiography. Values are means ± SD. *P < 0.05, **P < 0.01 vs. control; ^#^P < 0.05, ^##^P < 0.01 vs. DM+control.

Most parameters of cardiac function were monitored with hemodynamic measurements (**Figures 3A–C**) and echocardiography (**Figures 3D–F**). Left ventricular function was impaired in diabetic rats as shown with a significant decrease in dp/dt_max_, dp/dt_min_, FS%, and EF%, and an increase in LVEDP compared to non-diabetic controls. Cardiac output (CO), heart rate and LVESP in diabetic rats were not statistically different from non-diabetic controls (data not shown). After 6 months of rAAV-DCN treatment, diabetes-induced cardiac dysfunction was significantly improved, with an increase in dP/dt_max_, dP/dt_min_, FS% as well as EF% and a decrease in LVEDP, compared to the diabetic control group (**Figures 3A–F**).

### rAAV-DCN Attenuated Diabetes-Induced Myocardial Fibrosis *In Vivo* by Inhibition of TGFβ1 Pathway

**Figure 4:**
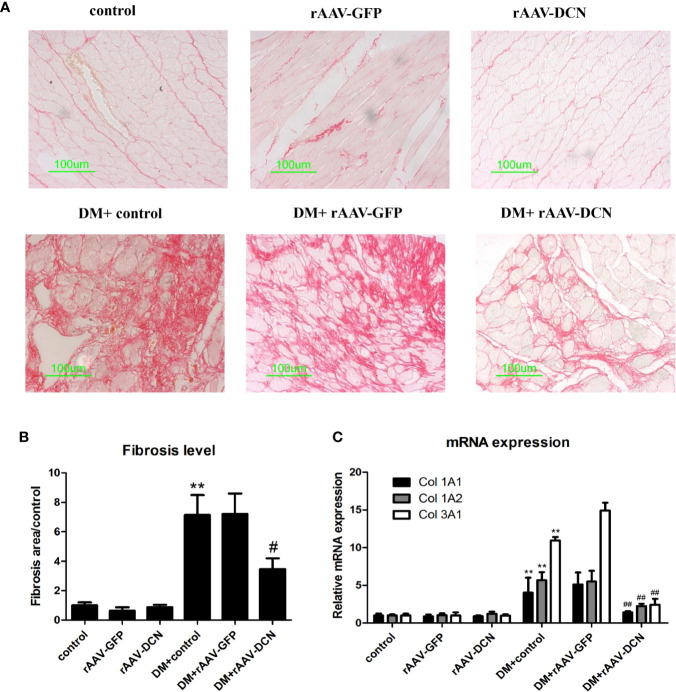
Decorin (DCN) overexpression attenuated myocardial fibrosis in diabetic rats. **(A)** Collagen deposition in cardiac tissues indicated by Sirius red staining. **(B)** The ratio of the fibrosis area evaluated with image pro plus software; **(C)** The mRNA levels of collagen type 1 (Col 1A1, Col 1A2) and type 3 (Col 3A1) assessed by real-time RT-PCR. GAPDH was served as housekeeping gene. Values are means ± SD. *P < 0.05, **P < 0.01 vs. control; ^#^P < 0.05, ^##^P < 0.01 vs. DM+control.

As we all known, the pathogenesis of DCM is myocardial remodeling. To investigate whether overexpression of DCN attenuated myocardial remodeling in diabetic rats, the cardiac fibrosis level was evaluated by Sirius Red staining. As shown in **Figures 4A, B**, markedly increased amount of red-stained area (collagen) was observed in diabetic rats compared with non-diabetic controls. rAAV-DCN treatment in diabetic rats significantly attenuated the collagen deposition in cardiac tissues, compared to the diabetic rats (**Figures 4A, B**). Furthermore, mRNA levels of Col 1A1, Col 1A2, and Col 3A1 in cardiac tissues were validated by real-time RT-PCR (**Figure 4C**). Results showed that diabetes induced significantly increased of mRNA levels of collagen type 1 and type 3 compared with controls. In contrary, rAAV-DCN treatment resulted in a significant reduction of mRNA level in collagens type 1 (Col 1A1, Col 1A2) and type 3 (Col 3A1) in diabetic rats (**Figure 4C**).

**Figure 5:**
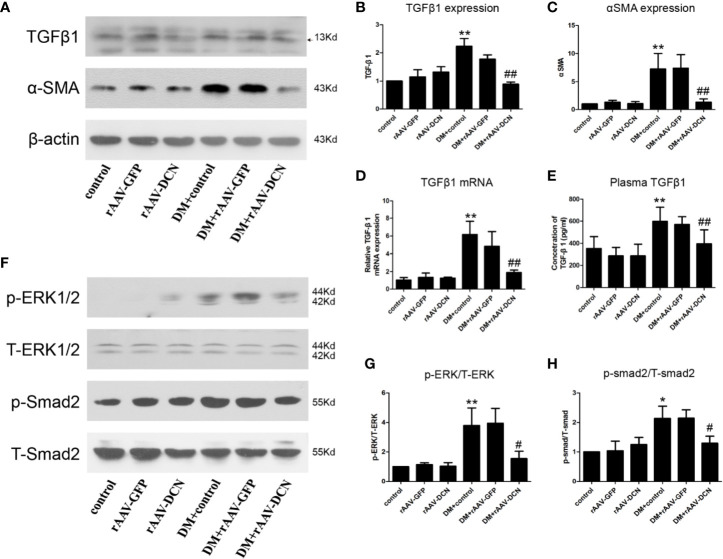
TGFβ pathway was involved in the protection of decorin (DCN) against myocardial fibrosis in diabetes. **(A–C)** The protein level of TGFβ1 **(A, B)** and α-SMA **(A, C)** detected by western blot; **(D)** The mRNA expression of TGFβ1 in heart tissue; **(E)** The concentration of TGFβ1 in plasma; **(F–H)** the phosphorylation level of ERK1/2 **(F, G)** and smad2 **(F, H)**. Values are means ± SD. *P < 0.05, **P < 0.01 vs. control vs. control; ^#^P < 0.05, ^##^P < 0.01 vs. DM+control.

Furthermore, we detected the protein of TGFβ1 and α-SMA in heart tissue (**Figures 5A–C**). Results showed that compared to control, the expression of TGFβ1 and α-SMA were elevated in DM group, and decreased by overexpression of DCN. Meanwhile, the mRNA of TGFβ1 and its concentration in plasma were evaluated, which was similar to that of protein expression among groups (**Figures 5D, E**). Besides, the pathway including canonical (smad2) and non-canonical (ERK1/2) were assessed. Results showed that the phosphorylation level of smad2 and ERK1/2 were increased in DM and ameliorated by overexpression of DCN (**Figures 5F–H**). It meant that overexpression of DCN attenuated diabetes-induced myocardial fibrosis by inhibition of TGFβ1 pathway.

### rAAV-DCN Attenuated Diabetes-Induced Myocardial Inflammation

**Figure 6:**
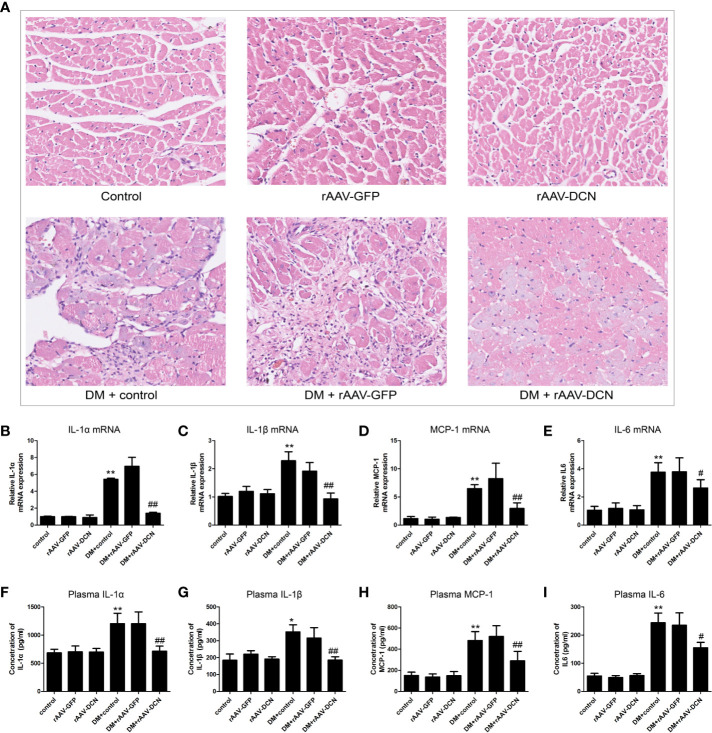
Overexpression of decorin (DCN) reduced inflammation in the diabetic animals. **(A)** HE staining of heart sections; **(B–E)** mRNA level of IL-1α **(B)**, IL-1β **(C)**, MCP-1 **(D)**, and IL-6 **(E)** were determined by real-time RT-PCR, respectively; **(F–I)** Concentration of plasma IL-1α **(F)**, IL-1β **(G)**, MCP-1 **(H)**, and IL-6 **(I)** were determined by ELISA, respectively. Values are means ± SD. *P < 0.05, **P < 0.01 vs. control vs. control; ^#^P < 0.05, ^##^P < 0.01 vs. DM+control.

HE staining was carried out in the present study (**Figure 6A**). Results showed that DM induced the inflammatory cells infiltration and reduction of cardiomyocytes, which could be ameliorated by DCN gene delivery (**Figure 6A**, **Supplementary Figures 3A, B**). To investigate the effects of rAAV-DCN treatment on diabetes-induced myocardial inflammation, real-time RT PCR was carried out with primers specific for the pro-inflammation cytokines, including IL-1α, IL-1β, mcp-1, and IL-6. As shown in **Figures 6B–I**, results turned out that expression of pro-inflammation cytokines were significantly increased in diabetic rats, compared to that of non-diabetic controls. However, mRNA levels of IL-1α, IL-1β, mcp-1, and IL-6 in rAAV-DCN-treated diabetic rats were significantly decreased (**Figures 6B–E**). Furthermore, plasma levels of IL-1α, IL-1β, mcp-1, and IL-6 were determined by ELISA. As expected, rAAV-DCN treatment significantly decreased the plasma levels of IL-1α, IL-1β, mcp-1, and IL-6 comparing with the diabetic group (**Figures 6F–I**). These data showed that rAAV-DCN treatment attenuated DM-induced myocardial inflammation.

### Mechanism of DCN Prevents Diabetic Induced Myocardial Inflammation

**Figure 7:**
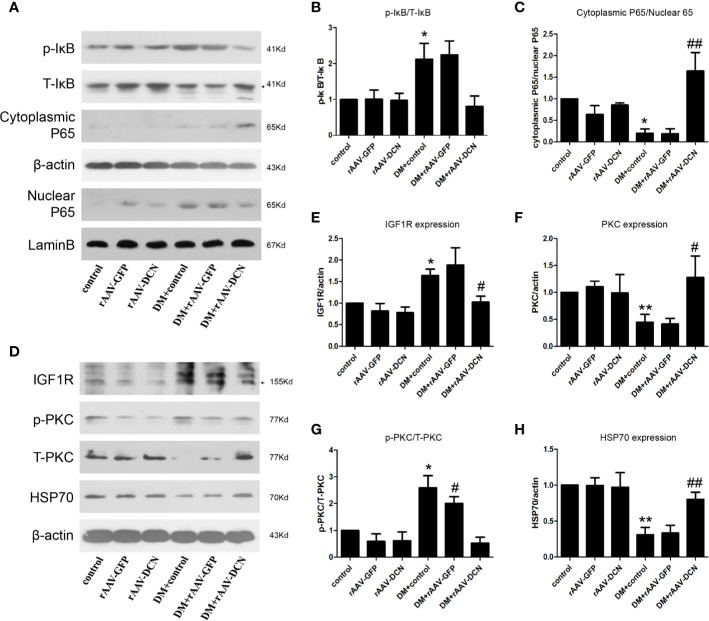
NF-κB and IGF-IR/PKCα/hsp70 pathway were involved in the protection of decorin (DCN) against diabetic cardiomyopathy (DCM). **(A–C)** The expression of p-IκB, T-IκB, cytoplasmic P65, and nuclear P65. β-actin were used for cytoplasmic control and lamin B were used for nuclear control. **(D–H)** the expression of IGFIR **(D, E)**, p-PKCα **(D, F)**, T-PKCα **(D, G)** as well as HSP70 **(D, H)** were determined by Western blots. Values are means ± SD. *P < 0.05, **P < 0.01 vs. control; ^#^P < 0.05, ^##^P < 0.01 vs. DM+control.

In the present study, we found that rAAV-DCN ameliorated DCM *via* inhibition of the expression of pro-inﬂammatory and pro-fibrotic cytokines. Thus, we investigated the influences of rAAV-DCN on NF-κB pathways. We found that DCN gene therapy resulted in not only a marked decrease in phosphorylated levels of IκB-α, but also a reduction in nuclear localization of p65 concomitantly in hearts of diabetic rats (**Figures 7A–C**). These data suggested that DCN overexpression prevented the hyper-activation of NF-κB caused by diabetes. Furthermore, we investigated the expression of IGF-IR, p-PKCα, PKCα, and Hsp70, which related with NF-κB signaling pathways. We found that DCN overexpression significantly down-regulated the IGF-IR, p-PKCα level but increased total PKCα and Hsp70 expression in the hearts of diabetic rats (**Figures 7D–H**).

## Discussion

In the present study, combination of low dose STZ and high fat diet was used to induce the DCM, and the effects of rAAV-mediated DCN therapy was evaluated in DCM. As shown above, the heart function was damaged in the diabetic rats, and aggregated by the overexpression of DCN. Besides, both fibrosis and inflammation was ameliorated by DCN overexpression in DCM, which was mediated by IGF1R/PKCα/HSP70 and TGFβ1 pathway.

As a key pathological feature of DCM (4), cardiac ﬁbrosis is characterized by destruction of cardiomyocytes and accumulation of ﬁbrillar collagen (17). The collagen accumulation will increase myocardial stiffness that leads to diastolic dysfunction and systolic dysfunction. In the present study, we found that DCN gene therapy resulted in regression of cardiac ﬁbrosis and signiﬁcantly improved cardiac function. In the progression of DCM, TGFβ1 is an important pro-fibrotic cytokine, which result in ventricular remodeling and cardiac dysfunction. It is known that DCN is a nature inhibitor for TGF-β1 (18). Therefore, we evaluated the expression of TGF-β1 in the circulation. Encouragingly, our study confirmed the inhibiting effects of DCN on TGFβ1. Besides, Yan et al. reported that DCN inhibited the TGFβ through the canonical (smad2) pathway, which was similar to our results. Moreover, the present study showed that the non-canonical pathway (ERK1/2) of TGFβ were also inhibited by DCN in DCM.

The major complication of fibrosis, inflammation is also an important role in DCM (19). Previous studies reported that TGFβ1 was an activator for NFκB (20), which is involved in the inﬂammatory process. Blockade of NF-κB may effectively suppress expression of pro-inﬂammatory and pro-ﬁbrogenic genes (21). NF-κB is activated in diabetes and may play an important role in the development of cardiovascular complications in diabetes (22). We investigated that whether DCN play the protective role in diabetes not only through reduction of TGF-β1 but also by suppressing NF-κB. Results turned out that the NFκB pathway was activated in DCM, accompanied by the increase of the pro-inflammatory cytokine, while all these could be inhibited by overexpression of DCN.

2017, we firstly reported the protective effects of DCN against DCM *via* promoting angiogenesis (13). However, the effects of DCN on cardiomyocyte is still unknown, which was revealed in the present study. It was showed that DCN ameliorated DCM by inhibition of fibrosis and inflammation, and the mechanism underlying was explored. Hsp70 family is one of the most abundant stress proteins in the human body. There are two very similar 70-kDa heat shock proteins in the mammalian cytoplasm: constitutively expressed cytoplasmic 73-kDa-mass heat shock protein (Hsc-73) and inducible cytoplasmic 72-kDa-mass heat shock protein (Hsp-72, also named as Hsp70) (23). Previous study showed that hsp70 is cytoprotective stress protein and has the anti-inﬂammatory and anti-fibrosis activity by inhibiting NF-κB activity and the production of TGF-β1.

Heat shock (HS) treatment induced high-level expression of Hsp70, which inhibited nuclear translocation and activity of NF-κB and suppressed the degradation of IκB and the accumulation of phosphorylated IκB (24–26). Chen et al. found that HS treatment reduced ANG II-induced leukocyte inﬁltration, perivascular and interstitial inﬂammation, and ﬁbrosis in the heart tissues *via* the modulation of NF-κB activity (25). In addition, Kavanagh et al. found that type 2 diabetic monkeys had significantly lower HSP70 expression in circulation and liver tissues (27). Induction of Hsp70 may have a favorable impact on the microvascular complications of type 2 diabetes, and improve the insulin sensitivity of type 2 diabetics (28). In the present study, we found that rAAV-DCN significantly increases the expression of hsp70, along with TGF-β1 reduction and inactivation of NF-κB pathway in hearts of diabetic rats, which was consistent with previous research.

Furthermore, studies showed that PKCα plays a critical role in the expression of Hsp70. Coaxum et al. also found that PKCα is able to induce expression of the inducible form of HSP70 in H9c2 cells (29). The insulin-like growth factor receptor I (IGF-IR), a ligand activated tyrosine protein kinase, is highly homologous to the insulin receptor. Li et al. showed that overexpression and activation of IGF-IR can inhibit PKCα gene transcription (30). It has been reported that DCN could bound to the IGF-IR and resulted in its down-regulation (10, 31). In streptozotocin induced diabetes in DCN-/- mice, lack of DCN increased the rate of apoptosis, expression levels of the pro-inflammatory cytokines and the infiltration of mononuclear cells in diabetic kidneys, the mechanism may be associated with the overexpression of IGF-IR caused by DCN knockdown (10). These results indicate DCN may prevent cardiac fibrosis and inﬂammation by regulating the expression of IGF-IR/PKCα/hsp70 pathway. In the present study, we confirmed that delivering recombinant DCN resulted in the reduction of IGF-IR and induction of PKCα and hsp70 in hearts of diabetic rats.

However, there were also limitations in the present study. Firstly, the rAAV serotype 9 with CMV promotor was employed. This vector gained increasing popularity as delivery systems for cardiac therapeutic gene transfer, primarily due to their non-pathogenic, broadly tropic nature and high specificity to the heart. Pacak et al. reported that rAAV serotype 9 was able to transduce myocardium at 200-fold higher levels than rAAV serotype 2 (32). Even though, it also could transduce the gene expression in other tissues, for example the liver and skeletal muscle (32). This limitation was mainly due to the un-specific promotor (CMV promotor), which allowed a multiorgan expression of target gene. Fortunately, it has been reported that a novel rAAV 9 system employing the cardiac troponin T (cTnT) promoter showed cardiomyocyte-specific gene expression (33). It could be used in our future work. Besides, the vector was injected intravenously. As we known, AAV is a non-enveloped, single-stranded DNA virus with a small, icosahedral capsid of approximately 25 nm (34), which is hardly to across the barrier endothelium freely. In the present study, we observed that the target gene was upregulated by the delivery system, but the mechanism underlying AAV through barrier endothelium is remained unknown. It has been reported that the virus could penetrate barrier cells by transcytosis (35). Finally, some parameters of the cardiac function showed no significant difference, which might be due to the small sample size.

Collectively, our findings showed that DCN overexpression inhibited the fibrosis and inflammation in DCM through reduction of TGF-β1 and inactivation of NF-κB respectively, which may be mediated by IGF-IR/PKCα/hsp70 pathway. Therefore, DCN gene therapy is possibly a promising treatment modality to prevent DCM in the DM.

## Data Availability Statement

The datasets generated for this study are available on request to the corresponding authors.

## Ethics Statement

The animal study was reviewed and approved by the committee on the Ethics of Animal Experiments of the Animal Research Committee of Tongji Medical College.

## Author Contributions

FC and JL conceived and designed the experiments, performed the experiments, analyzed the data, and contributed to the writing of the manuscript. YZ and MH conceived and designed the experiments, performed the experiments, analyzed the data, and contributed analysis tools. HH and JW performed the experiments. CC conceived and designed the experiments and participated in the writing of the manuscript. JT conceived and designed the experiments and contributed to the writing of the manuscript. DW conceived and designed the experiments. All authors contributed to the article and approved the submitted version.

## Funding

This work was supported by the National Natural Science Foundation of China (No. 30871068 and 81400334) and the Wuhan Science and Technology Bureau (No. 2014060101010037).

## Conflict of Interest

The authors declare that the research was conducted in the absence of any commercial or financial relationships that could be construed as a potential conflict of interest.

## Supplementary Material

Supplementary Figure 1 The efficiency of rAAV-GFP in the heart. **(A)** the expression of GFP proteins in the heart, **(B)** the expression of GFP in the heart of rat received rAAV-GFP. Green is represented of GFP; blue, DAPI for nucleus.

Supplementary Figure 2 Effect of DCN on plasma lipids profiles. **(A)** Plasma triglyceride (TG), **(B)** total cholesterol (TC), **(C)** low-density lipoprotein (LDL) and **(D)** high-density lipoprotein (HDL) levels in the six groups were measured. Values are means ± SD. *, P<0.05 vs. control groups; #, P<0.05 vs. DM+control.

Supplementary Figure 3 Quantification of the heart HE staining. **(A)** the inflammatory cell counts; **(B)** the cardiomyocyte counts. Values are means ± SD. *, P<0.05 vs. control groups; #, P<0.05 vs. DM+control.
