# Supplementary figures and images for: Cardioprotective Effect of Decorin in Type 2 Diabetes
Source: Front Endocrinol (Lausanne). 2020 Dec 7;11:479258. doi: 10.3389/fendo.2020.479258 (PMC7750479; doi:10.3389/fendo.2020.479258)

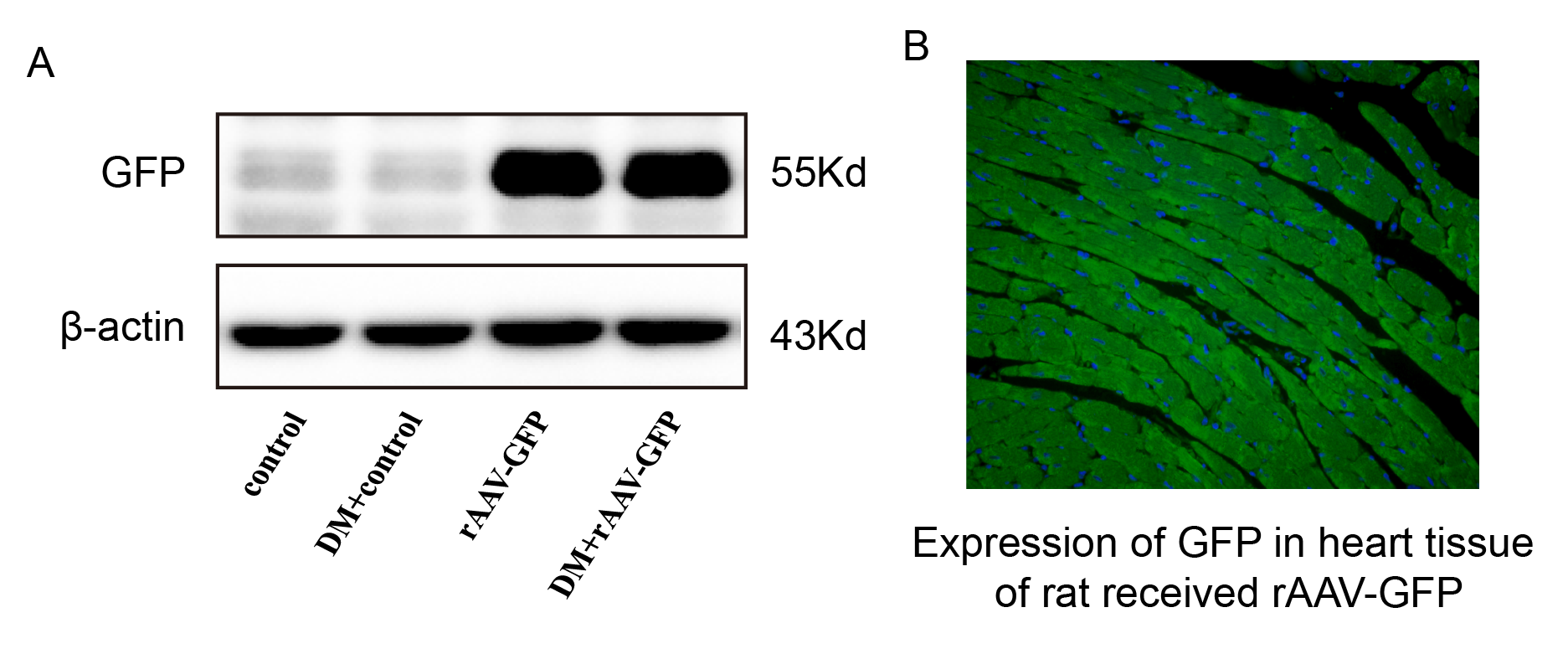

Supplement: Supplementary Figure 1 — The efficiency of rAAV-GFP in the heart. (A) the expression of GFP proteins in the heart, (B) the expression of GFP in the heart of rat received rAAV-GFP. Green is represented of GFP; blue, DAPI for nucleus. [file Image_1.tif]

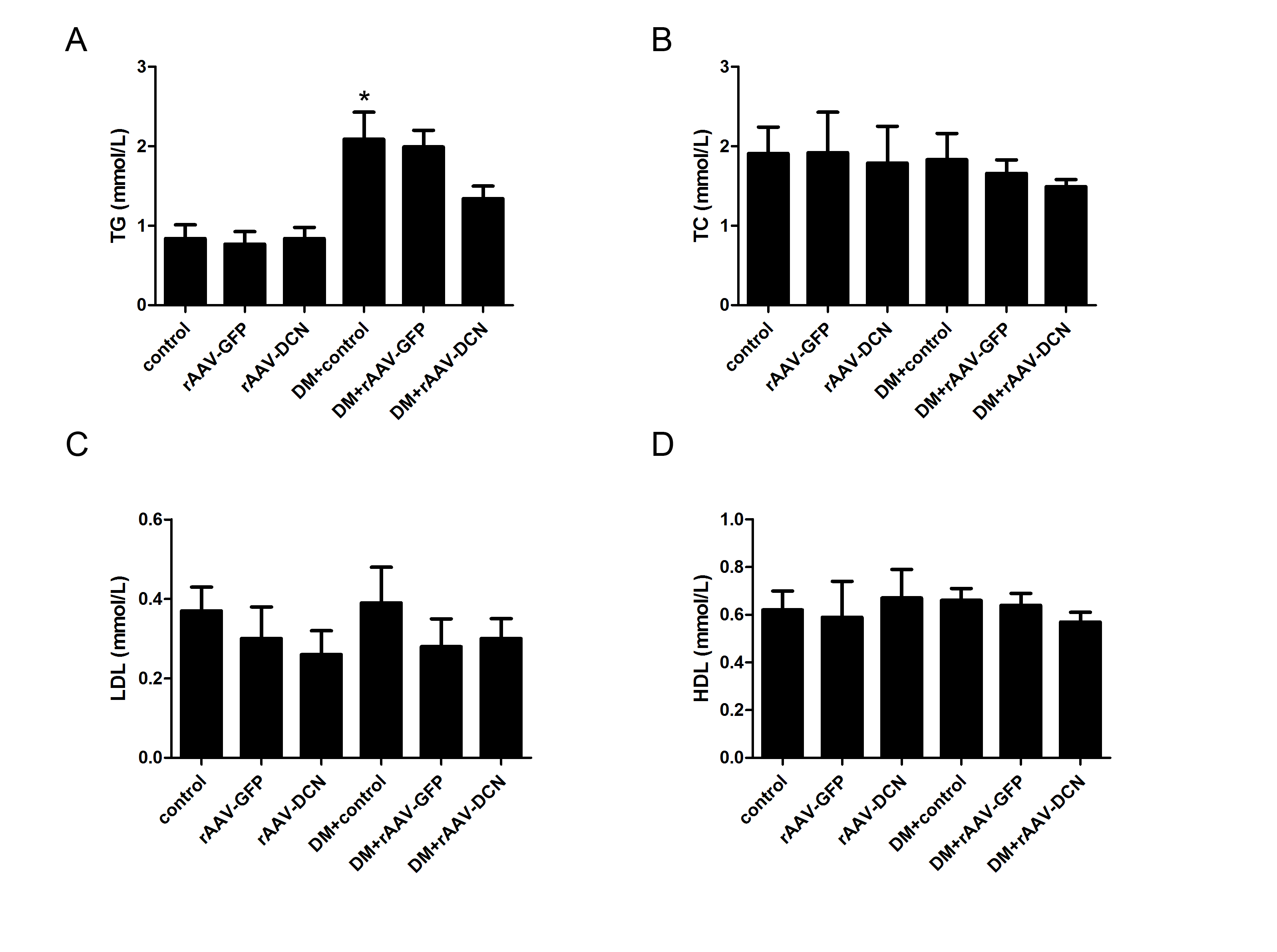

Supplement: Supplementary Figure 2 — Effect of DCN on plasma lipids profiles. (A) Plasma triglyceride (TG), (B) total cholesterol (TC), (C) low-density lipoprotein (LDL) and (D) high-density lipoprotein (HDL) levels in the six groups were measured. Values are means ± SD. *, P<0.05 vs. control groups; #, P<0.05 vs. DM+control. [file Image_2.tif]
