# Supplementary material for: Cardioprotective Effect of Decorin in Type 2 Diabetes
Source: Front Endocrinol (Lausanne). 2020 Dec 7;11:479258. doi: 10.3389/fendo.2020.479258 (PMC7750479; doi:10.3389/fendo.2020.479258)
Supplement: Supplementary file 4 [file Table_1.docx]

**Supplementary table 1.** The Primers used for real time RT-PCR.

| **Gene Name** | **Forward** | **Reverse** |
| --- | --- | --- |
| IL 1α | 5’-TGTTGCTGAAGGAGTTGCCAG-3’ | 5’-CCCGACTTTGTTCTTTGGTGG-3’ |
| IL 1β | 5’-TGGTGTGTGACGTTCCCATT-3’ | 5’-CAGCACGAGGCTTTTTTGTTG-3’ |
| IL 6 | 5’ AGCGATGATGCACTGTCAGA 3’ | 5’ GGAACTCCAGAAGACCAGAGC 3’ |
| MCP-1 | 5’-GATCGGAACCAAATGAGATCAG-3’ | 5’-GTGGAAAAGGTAGTGGATGC-3’ |
| TGF β1 | 5’-TGGACCGCAACAACGCAATCTA-3’ | 5’-CACCTCGACGTTTGGGACTGATC-3’ |
| Col 1A1 | 5’-GAGCGGAGAGTACTGGATCG-3’ | 5’-GAT TGGGATGGAGGGAGTTT-3’ |
| Col 1A2 | 5’-GAGACCCTTCTCACTCCTG-3’ | 5’-TCCAGAGGTGCAATGTCAAG-3’ |
| Col 3A1 | 5’-GGCTGCAAGATGGATGCTAT-3’ | 5’-TTTTGTTTTGCTGGGGTTTC-3’ |
| DCN | 5’-AGTGTTCTGATTTGGGTCTGGA-3’ | 5’-CAAGAATCAATGCGTGAAGGTT-3’ |
| GAPDH | 5’-TGCTATGTTGCCCTAGACTTCC-3’ | 5’-GTTGGCATAGGTCTTTACGG-3’ |
