# Supplementary material for: Cardioprotective Effect of Decorin in Type 2 Diabetes
Source: Front Endocrinol (Lausanne). 2020 Dec 7;11:479258. doi: 10.3389/fendo.2020.479258 (PMC7750479; doi:10.3389/fendo.2020.479258)
Supplement: Supplementary file 5 [file Table_2.docx]

**Supplementary table 2.** The information of the antibodies used.

| **Antibody** | **Specific** | **Type** | **Dilution** | **Source** |
| --- | --- | --- | --- | --- |
| Decorin | Total decorin | Mono- | 1:500 | Santa cruz |
| GFP | Total | Mono- | 1:1000 | Abclonal |
| β-actin | Total | Mono- | 1:1000 | Santa cruz |
| p-samd2 | Phosphorylated smad2 at S255 | Mono- | 1:1000 | Abcam |
| T-smad2 | Total smad2 | Mono- | 1:1000 | Abcam |
| p-ERK1/2 | Phosphorylated ERK1 at T202 and Y204; phosphorylated ERK2 at T185 and Y187 | Mono- | 1:1000 | Abcam |
| T-ERK1/2 | Total ERK1 and ERK2 | Poly- | 1:1000 | Abcam |
| p-IκB | Phosphorylated IκB at S32 | Mono- | 1:1000 | Santa cruz |
| T-IκB | Total | Mono- | 1:1000 | Santa cruz |
| P65 | Total P65 unit | Mono- | 1:1000 | Santa cruz |
| Lamin B | Total | Mono- | 1:1000 | Santa cruz |
| IGF1R | Total IGF1R α unit | Poly- | 1:1000 | Boster Biological |
| p-PKC | Phosphorylated PKC at T514 | Mono- | 1:2000 | Abcam |
| T-PKC | Total PKC | Mono- | 1:1000 | Abcam |
| HSP70 | Total | Mono- | 1:1000 | Boster Biological |
| TGF-β1 | Total | Mono- | 1:1000 | Santa cruz |
| α-SMA | Total | Mono- | 1:1000 | Santa cruz |
